# JS-K, a glutathione/glutathione S-transferase-activated nitric oxide releasing prodrug inhibits androgen receptor and WNT-signaling in prostate cancer cells

**DOI:** 10.1186/1471-2407-12-130

**Published:** 2012-03-30

**Authors:** Martin Laschak, Klaus-Dieter Spindler, Andres J Schrader, Andrea Hessenauer, Wolfgang Streicher, Mark Schrader, Marcus V Cronauer

**Affiliations:** 1 Institute for General Zoology and Endocrinology, Ulm University, Albert Einstein Allee 23, 89069 Ulm, Germany; 2 Department of Urology, Ulm University, Prittwitzstrasse 43, 89075, Ulm, Germany

## Abstract

**Background:**

Nitric oxide (NO) and its oxidative reaction products have been repeatedly shown to block steroid receptor function via nitrosation of zinc finger structures in the DNA-binding domain (DBD). In consequence NO-donors could be of special interest for the treatment of deregulated androgen receptor(AR)-signaling in castration resistant prostate cancer (CRPC).

**Methods:**

Prostate cancer (PCa) cells were treated with JS-K, a diazeniumdiolate derivate capable of generating large amounts of intracellular NO following activation by glutathione S-transferase. Generation of NO was determined indirectly by the detection of nitrate in tissue culture medium or by immunodetection of nitrotyrosine in the cytoplasm. Effects of JS-K on intracellular AR-levels were determined by western blotting. AR-dimerization was analyzed by mammalian two hybrid assay, nuclear translocation of the AR was visualized in PCa cells transfected with a green fluorescent AR-Eos fusion protein using fluorescence microscopy. Modulation of AR- and WNT-signalling by JS-K was investigated using reporter gene assays. Tumor cell proliferation following JS-K treatment was measured by MTT-Assay.

**Results:**

The NO-releasing compound JS-K was shown to inhibit AR-mediated reporter gene activity in 22Rv1 CRPC cells. Inhibition of AR signaling was neither due to an inhibition of nuclear import nor to a reduction in AR-dimerization. In contrast to previously tested NO-donors, JS-K was able to reduce the intracellular concentration of functional AR. This could be attributed to the generation of extremely high intracellular levels of the free radical NO as demonstrated indirectly by high levels of nitrotyrosine in JS-K treated cells. Moreover, JS-K diminished WNT-signaling in AR-positive 22Rv1 cells. In line with these observations, castration resistant 22Rv1 cells were found to be more susceptible to the growth inhibitory effects of JS-K than the androgen dependent LNCaP which do not exhibit an active WNT-signaling pathway.

**Conclusions:**

Our results suggest that small molecules able to inhibit WNT- and AR-signaling via NO-release represent a promising platform for the development of new compounds for the treatment of CRPC.

## Background

Nitric oxide (NO), a free radical gas, is a pleiotropic molecule critical to a number of physiological and pathological processes. NO-releasing drugs are a growing class of promising new therapeutics with applications in a large variety of diseases like cardiovascular and respiratory disorders, osteoporosis, Alzheimer's disease, inflammatory lesions and urinary incontinence [1-4]. Moreover there is increasing evidence that NO donors could have potential in the prevention and therapy of various malignant tumors like myeloma, breast cancer, ovarian cancer, pancreatic cancer or prostate cancer (PCa) [5-11].

PCa is the most commonly diagnosed neoplasm in elderly men and the second cause of cancer related deaths in the Western world [12]. Current treatment for advanced PCa is mainly based on androgen ablation therapies like orchiectomy, systemic administration of LHRH analog/blocker or anti-androgens. Unfortunately, the benefit of androgen ablation is only transitory. Within a few years many PCa progress to a state of the disease termed castration resistant prostate cancer (CPRC) where tumor cells grow and survive under subphysiological levels of androgens [13]. Although *in vitro *the development of a castration resistant phenotype is mostly based on the loss of the AR in PCa cells, several clinical studies demonstrated that the AR is rarely lost in CRPC cells *in vivo *[14-16]. Indeed, CRPC cells continue to depend on AR-signalling but bypass the requirements for physiological levels of circulating androgens. Various molecular mechanisms that promote AR-dependent growth of CRPC cells growing under androgen deprived conditions have been identified: over-expression/amplification of the AR (hypersensitive pathway), AR mutations that broaden ligand specificity (promiscious pathway), AR-activation by non steroid ligands like growth factors or cytokines (outlaw pathway) [17] as well as the expression of C-terminally truncated AR variants lacking vast parts of the ligand binding domain (LBD). These AR-variants, termed ARΔLBD, are either products of alternative splicing (AR-V), point mutations leading to premature stop codons or proteolytic cleavage of the AR protein [18-21]. In contrast to a full length AR which is activated upon androgenic stimuli, previous *in vitro *studies were able to show that most ARΔLBDs, devoid of a ligand binding domain, are constitutively active [18-21]. As ARΔLBDs lack most parts of the LBD situated in the C-terminus of the AR, they are insensitive to any form of androgen ablation.

Nitric oxide (NO) and its oxidative reaction products have been repeatedly shown to block nuclear receptors via nitrosation of their zinc finger structures in the DNA-binding domain (DBD). The DBD is an essential part of functional full length AR as well as the constitutively active ARΔLBDs. In consequence NO-donors could be of special interest for the treatment of deregulated AR-signalling in CRPC cells. Inhibition of AR-functions following treatment with the long living spontaneous NO-donor (Z)-1-[N-(2-Aminoethyl)-N-(2-ammonioethyl)amino]diazen-1-ium-1,2-diolat (DETA/NO) has recently been demonstrated [22]. However, due to the short half-life of the free radical NO in cell culture medium, relatively high concentrations of DETA/NO were necessary to induce effects on intracellular nuclear receptors. In order to generate large amounts of intracellular NO, new compounds, able to deliver NO in a spatially controllable manner, are needed. In our study we used the non-ionic, nitroaromatic diazeniumdiolate JS-K, a glutathione S-transferase(GST)-activated NO-prodrug able to generate high intracellular levels of NO in the micromolar range [23,24].

Studying the effects of JS-K on AR- and WNT-signalling in the AR-positive PCa cell lines 22Rv1 and LNCaP we were able to demonstrate that only a 100-fold lower concentration of the GST-activated JS-K are necessary to reach comparable biological effects in PCa cells than the commonly used DETA/NO that spontaneously generates NO in physiological fluids. Our data show that NO-prodrugs, capable of generating large intracellular amounts of NO, may be suitable for the treatment of advanced prostate cancer.

## Methods

### Plasmids, antibodies, chemicals

Probasin promoter driven luciferase-reporter plasmid (pGL3E-Probasin) was kindly provided by Prof. Dr. Z. Culig (Innsbruck, Austria). The pARt1EosFP expression plasmid coding for a green fluorescent AR-Eos fusion protein (AR-EosFP) was a gift from Prof. F. Oswald (Ulm, Germany). The CMV-promoter driven β-catenin expression plasmid pbCAT, containing human β-catenin cDNA with an activating S33Y mutation was provided by Dr. H. Clevers (Utrecht, The Netherlands). TCF-reporter plasmids pTopFlash (TOP) and pFopFlash (FOP), containing three copies of wild-type or mutant TCF-binding sites upstream of a thymidine kinase minimal promoter driving a luciferase gene, were purchased from Upstate Biotechnology (Lake Placid, NY, USA). *Renilla reniformis *luciferase reporter plasmid pRL-tk-LUC was a product of Promega (Mannheim, Germany). Mouse monoclonal antibodies directed against beta actin (ab8224) and nitrotyrosine (ab7048) were products of Abcam (Cambridge, UK). Mouse monoclonal antibody AR441 directed against the N-terminus of the AR was purchased from Dako (Hamburg, Germany). The NO-donors O2-(2,4-Dinitrophenyl)-1-[(4-ethoxycarbo-nyl)piperazin-1-yl]diazen-1-ium-1,2-diolate (JS-K), (Z)-1-[N-(2-Aminoethyl)-N-(2-ammonioethyl)amino]diazen-1-ium-1,2-diolate (DETA/NO) and the androgen dihydrotestosterone (DHT) were a products of Sigma Aldrich (Taufkirchen, Germany).

### Cell culture

22Rv1, LNCaP, DU-145 and PC-3 cells were purchased from the American Type Culture Collection (Manassas, VA, USA). LNCaP-SSR, a castration resistant LNCaP subline [25,26], was a generous gift from Prof. Martin Burchardt, Greifswald, Germany. 22Rv1, LNCaP, DU-145 and PC-3 were grown in RPMI-1640 (PAA Laboratories GmbH, Pasching, Austria) supplemented with 10% fetal bovine serum (FBS) and 1% Penicillin/Streptomycin (BioWest, Nuaille, France). LNCaP-SSR were routinely grown in RPMI-1640, 1% Penicillin/Streptomycin supplemented with 10% steroid free, dextran charcoal treated FBS (FBSdcc, BioWest, Nuaille, France) [26].

### Transfection and reporter gene assays

22Rv1 cells were grown on 24-well plates (Sarstedt, Nümbrecht, Germany). Transfections were performed using FuGene HD transfection reagent (Roche Diagnostics, Mannheim, Germany) according to the manufacturer's instructions. AR- and WNT-specific reporter gene assays were performed as recently described [22,27]. In brief: (1) AR-signalling: Cells were transfected with 200 ng/well of the AR-dependent reporter-plasmid pGL3E-Probasin. The pRL-tk-LUC vector coding for a Renilla luciferase under control of a constitutively active thymidine kinase promoter was co-transfected (80 ng/well) to correct for transfection efficiency. After transfection cells were grown in RPMI 1640 with 5% dextran charcoal treated steroid free, dextran charcoal treated FBS (FBSdcc; Biowest, Nuaillé, France) and treated with 5 nM DHT and different concentrations of JS-K. (2) WNT-signalling: Luciferase reporter plasmids pTopFlash (250 ng/well) and pFopFlash (250 ng/well) were mixed either with pbCAT expression vector (250 ng/well) or insert-free vector (250 ng/well). pRL-tk-luc (80 ng/well) was co-transfected to correct for transfection efficiency. After an incubation period of 12 h in RPMI-1640 with 5% FBSdcc, medium was changed to RPMI-1640 containing 2.5% FBSdcc with increasing concentrations of JS-K.

AR- and WNT-reporter activities were analyzed after 24 hours using the Dual Luciferase Reporter Assay System (Promega, Mannheim, Germany). Experiments were repeated at least three times and performed in triplicates, unless stated otherwise.

### Mammalian two hybrid assay

To investigate the dimerization of the AR we used the CheckMate/FlexiVector Mammalian Two-Hybrid System from Promega (Mannheim, Germany). VP16 activation domain (pFN10A-AR) and Gal4 DNA binding domain tagged androgen receptor fusion constructs (pFN11A-AR) were cloned according to the supplier's suggestions by using pSG5-AR as template. DU-145 and PC-3 cells were grown in 24-well plates and co-transfected with 150 ng/well of pFN10A-AR, pFN11A-AR and the Gal4 reporter construct pGL4.31. After transfection, cells were cultured and luciferase activities were determined as recently described [28].

### Nuclear Translocation assay

Nuclear translocation of the AR was analyzed in AR-negative DU-145 and PC-3 cells transfected with the green fluorescent AR-EosFP [22,29]. Therefore, prostate cancer cells were transfected in a 24-well plate with pAR-t1EosFP (0.25 μg/well) for 4 hours in RPMI and allowed to grow for another 24 hours in RPMI 1640 supplemented with 5% FBSdcc and antibiotics. Thereafter, cells were grown in RPMI supplemented with 2.5% FBSdcc in the presence/absence of 5 nM DHT and JS-K for 6 hours. Localization of the AR-fusion protein was subsequently analyzed by fluorescence microscopy.

### Western Blot analysis

Proteins were extracted from cells using RIPA buffer as recently described [30]. Proteins (15 μg lysate) were separated by Sodium dodecyl sulfate polyacrylamide gel electrophoresis (SDS-Page). Subsequently proteins were transferred onto a nitrocellulose membrane (BioTraceNT, Pall Life Sciences, Dreieich, Germany) by tank blotting (transfer buffer: 20 mM Tris/HCl, pH 8.7, 150 mM glycine and 20% (v/v) methanol). Membranes were blocked in phosphate buffered saline with 0.1% Tween20 (v/v) (PBS-T) and 5% BSA (w/v) for 1 hour at room temperature. The membrane was incubated with the primary antibody in PBS-T with 1% BSA over night at 4°C. All primary antibodies were used in a dilution of 1:1000, with the exception of the beta-actin antibody, which was diluted 1:20000. The membrane was washed with PBS-T three times before incubating with the peroxidase-coupled secondary antibody in a dilution of 1:2000 in PBS-T with 1% BSA. Signals were visualized by the SuperSignal West Pico Chemiluminescent Substrate from Pierce (Rockford, USA).

### Determination of NO

NO was determined indirectly by the photometric measurement of nitrite according to Green et al. [31]

### Immunocytochemistry

For immunocytochemistry 22Rv1 cells were cultured on glass coverslips (BD Falcon Culture Slides, BD Biosciences) washed (2 × 5 min) with PBS and fixed with 3.7% paraformaldehyd for 15 min at room temperature, washed (3 × 10 min) in PBS and permeabilized in 0.2% Triton X-100 with 1% BSA in PBS for 5 min at room temperature. The cells were washed (3 × 10 min) with PBS containing 1% BSA. Cells were then incubated with the monoclonal anti-tyrosine antibody (1:25 in PBS, 1% BSA for 30 min at 37°C, washed (3 × 10 min and incubated with the mouse monoclonal anti-goat antibody labeled with Alexa Fluor 488 (Invitrogen, Karlsruhe, Germany, 1:100) for 30 min at 37°C. Cells were then washed (3 × 10 min), the nuclei stained with DAPI (2.5 μg/ml in PBS) for 15 min, washed (3 × 10 min) and embedded with glycine and polyvinyle alcohol 4-88 (Fluka Analytical, Deisenhofen, Germany).

### Cell viability

Cell viability was determined by means of a colorimetric MTT-assay measuring the reduction of the water soluble tetrazolium bromide into its insoluble formazan derivative by functional mitochondria [32,33].

### Statistical Analysis

Data are reported as means ± standard deviation. Analysis was performed with Student's T-test (two tailed for independent samples) with p < 0.05 considered as significant.

## Results

### JS-K induces nitrosation of tyrosine residues in cellular proteins

**Figure 1:**
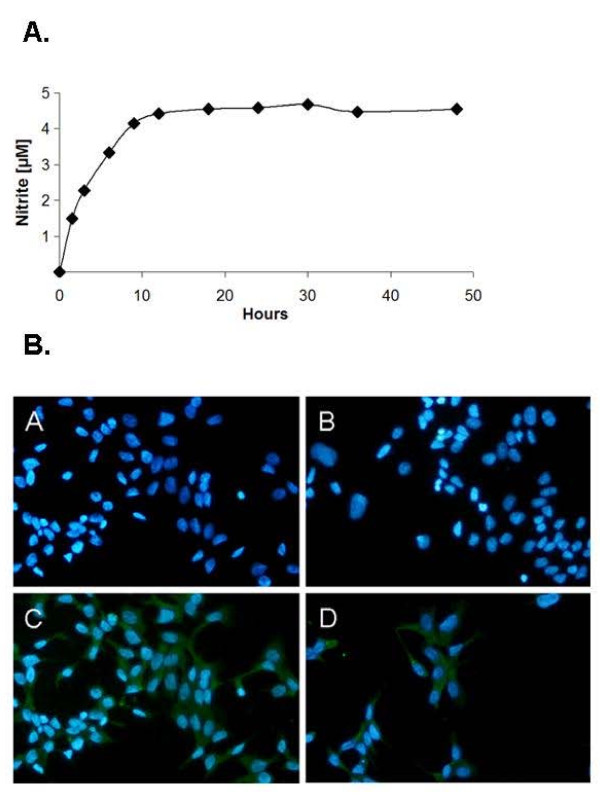
**Release of NO from JS-K**. (A) Generation of NO from JS-K in RPMI-1640 medium containing 10% fetal bovine serum was determined indirectly by photorimetric detection of nitrite according to Green et al. [31]. (B) Detection of nitrotyrosine by fluorescence microscopy in 22Rv1 cells: Generation of intracellular NO was detected indirectly by detection of nitrotyrosine (green). Cell nuclei were stained with DAPI. [A] untreated cells, [B] 2 μM JS-K_neg_, [C] 2 μM JS-K, [D] 200 μM DETA/NO.

In a previous paper NO generated by DETA/NO was shown to nitrosate tyrosine residues in proteins to nitrotyrosine [22]. Therefore, we tested JS-K's ability to nitrosate proteins via intracellular NO-release. In order to generate a control substance without the NO-releasing moiety, we took advantage of the observation that sera of patients were repeatedly shown to possess low GST-activity [34,35]. In our experiment 5 μM JS-K was incubated at 37°C in RPMI-1640 supplemented with 10% FBS. Generation of NO in the medium was measured indirectly by the determination of nitrite. Under these conditions maximal medium nitrite levels were detectable after nine to twelve hours (Figure 1A). The remaining inactive JS-K metabolites S-(2, 4-Dinitrophenyl)-glutathione and 4-carboxy-piperazine [23] were subsequently used as a negative control and termed JS-K_neg_.

Incubation with 2 μM JS-K as well as 200 μM DETA/NO (serving as positive control) led to a nitrosation of intracellular proteins as shown by fluorescescence microscopy of nitrotyrosine residues (Figure 1B). In contrast, neither untreated cells nor cells treated with the control solution JS-K_neg _harbouring the inactive JS-K metabolites, showed any nitrotyrosine staining (Figure 1B).

### JS-K inhibits AR-mediated genomic function in 22Rv1 cells

**Figure 2:**
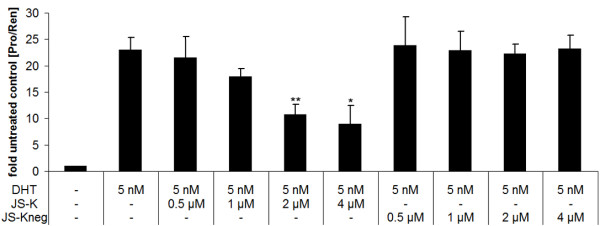
**JS-K inhibits AR-dependent reportergene activity in 22Rv1 cells**. Cells were cotransfected with a probasin reporter gene plasmid and a Renilla luciferase plasmid serving as transfection control. Subsequently cells were grown in presence/absence of 5 nM DHT. Reportergene activity was determined using the Dual Luciferase Assay System from Promega. Data are presented as fold of untreated controls ± standard deviation (SD). Results are mean values of three independent experiments performed in quadruplicates: *p < 0.05; **p < 0.01.

To test whether JS-K influences the transcriptional activity of the AR, we transiently co-transfected AR-positive 22Rv1 human prostate cancer cells with pGL3E-Pro, an androgen-responsive probasin-promoter driven reporter plasmid, and pRL-tk-Luc, a *Renilla *luciferase plasmid under control of a constitutively active thymidine kinase promoter (control of transfection efficiency). Subsequently cells were incubated for 44 hours with 5 nM DHT and increasing concentrations of JS-K or JS-K_neg_. JS-K significantly inhibited DHT-induced AR-transactivation in a dose-dependent manner. Inhibition was already significant at a concentration of 2 μM JS-K. In contrast, JS-K_neg _was unable to diminish AR transcriptional activity even at higher concentrations (Figure 2).

### JS-K does not modulate dimerization or nuclear localization of the AR

**Figure 3:**
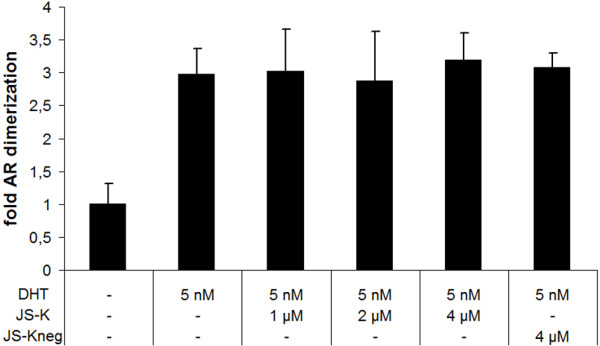
**JS-K does not inhibit AR-dimerization in presence of androgens**. AR-dimerization was determined in PC-3 cells using the CheckMate Mammalian Two-Hybrid System from Promega as described in Material and Methods. Results are mean values of four independent experiments performed in quadruplicates.

*In vitro *NO has been shown to repress vitamin D3 signalling through inhibition of vitamin D receptor (VDR) - retinoic X receptor (RXR)-heterodimerization [36]. To test whether JS-K can inhibit AR-homodimer formation *in vivo*, we performed an AR-specific mammalian two hybrid (M2H)-assay in AR-negative PC-3 and DU-145 cells (Figure 3, Additional File 1). As seen in Figure 3, JS-K was unable to repress DHT-induced AR-homodimerization at concentrations that were already sufficient to inhibit AR-transactivation (Figures 3 and 2).

**Figure 4:**
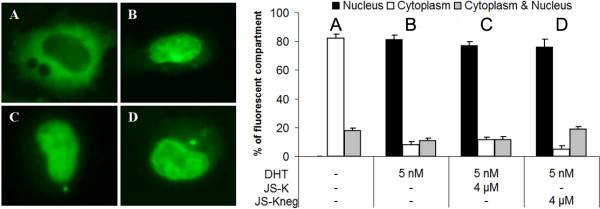
**NO does not influence hormone-induced nuclear translocation of the AR**. Prostate cancer cells (PC-3) expressing a green fluorescent AR-EosFP [37] were incubated for 6 hours in the absence/presence of DHT and JS-K/JS-Kneg. Intracellular localization of AR-EosFP was determined by fluorescence microscopy: (A) untreated controls, (B) 5 nM DHT, (C) 5 nM DHT and 4 μM JS-K, (D) 5 nM DHT and 4 μM JS-K_neg_. Bars: Data presented in % cellular localization ± SD. Results are mean values of three independent experiments performed in quadruplicates.

In order to test whether high NO concentrations are able to influence the nuclear translocation of the AR, we transfected the AR-negative PC-3 with a construct coding for a green fluorescent AR-Eos fusion protein (AR-EosFP) [22,37]. Subsequently cells were grown for six hours in the presence/absence of 5 nM DHT with or without JS-K or JS-K_neg_. Under hormone-free conditions the AR was predominantly located in the cytoplasm (> 80%) whereas in presence of DHT the AR was transported to the nucleus (Figure 4). As seen in Figure 4 addition of JS-K (final concentration 4 μM) was unable to reverse the nuclear localization of the AR in DHT treated PC-3 cells (82% ± 2% to 78 ± 2%). In a complementary experiment transfection of AR negative DU-145 cells with AR-EosFP yielded similar results (Additional File 2).

### JS-K leads to a down-regulation of the AR-protein

**Figure 5:**
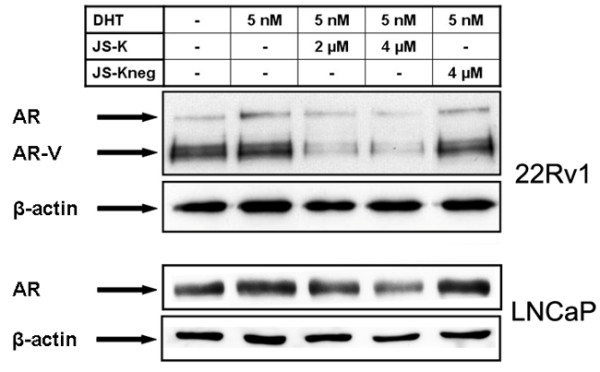
**Influence of JS-K and JS-Kneg on the androgen receptor concentration in human prostate cancer cells**. 22Rv1 and LNCaP cells were incubated for 30 hours with or without 5 nM dihydrotestosterone in the presence of JS-K or JS-K_neg_. Subsequently cells were lysed and separated by SDS-electrophoresis (15 μg protein per lane). Proteins were transferred onto a nitrocellulose membrane. AR and β-actin (loading control) were visualized by immunodetection.

In contrast to the commonly used spontaneous NO-donor DETA/NO which generates NO in aquaeous solutions, JS-K is able to generate high amounts of NO within target cells after enzymatic activation by GST. The profound differences in local NO-production prompted us to analyze the effects of JS-K on the AR-concentration in the prostate cancer line 22Rv1 and LNCaP. Both cell lines express functional AR-proteins. Whereas 22Rv1 cells express a 120 kDa AR-isoform as well as a C-terminally truncated, constitutively active 79 kDa AR-V splicing variant [38] the LNCaP cells express a full length AR (119 kDa) with a point mutation at position 877 (T877A) [39]. Treatment with JS-K for 30 hours diminished the amount of all AR-forms in 22Rv1 as well as in LNCaP cells (Figure 5). The downregulation of the AR in LNCaP was less pronounced than the dramatic decrease of AR and AR-V in 22Rv1 cells (Figure 5). In both cell lines incubation with JS-K_neg _had no influence on intracellular AR-levels (Figure 5).

### JS-K inhibits WNT/β-catenin signaling in 22Rv1 cells

Interaction of the canonical WNT/β-Catenin pathway with the AR is thought to promote progression of PCa to the terminal castrate-resistant stage [40]. Crosstalk between the canonical WNT and AR-pathways occurs at several levels: (a) β-Catenin interacts with the AR to increase its transcriptional activity [27], (b) WNT-signalling influences AR-signalling through its ability to regulate AR-mRNA as well as AR-stability [41].

**Figure 6:**
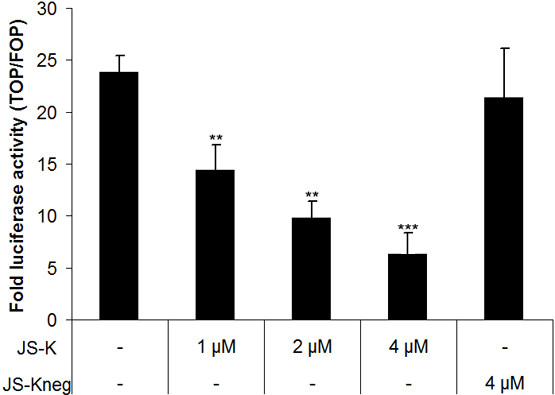
**JS-K diminishes WNT-signalling in 22Rv1 cells**. 22Rv1 cells were co-transfected with an expression vector for mutated, stabilized β-catenin together with either the TCF reporter construct TOP or FOP as recently described [25]. Data are presented as fold of untreated controls (TOP/FOP) ± SD, **p < 0.01, ***p < 0.001.

Several lines of evidence indicate that NO is involved in the regulation of the WNT/β-catenin pathway in various tumors [42-44]. A common approach to analyze the canonical WNT-pathway *in vitro *is the over-expression of mutated stabilized β-catenin (S33Y) that activate TCF/LEF-dependent reporter gene constructs [27]. As LNCaP cells are unable to activate β-catenin dependent reporter gene expression [27] we tested the effects of JS-K on the canonical WNT-pathway in 22Rv1 cells. As seen in Figure 6, JS-K was able to diminish β-catenin signalling in the CRPC cell line 22Rv1 in a dose dependent manner. Down-regulation of the canonical WNT-pathway was already significant at JS-K concentrations of 1 μM (reduction of transcriptional activity: 42 ± 8%, p < 0.01).

### Growth inhibitory effects of JS-K are most pronounced in castration resistant prostate cancer cells

Based on our observations we tested the antiproliferative effects of JS-K on prostate cancer cell lines. JS-K_neg _served as negative control. Cellular proliferation was assessed by means of a colorimetric MTT assay, measuring the reduction of tetrazolium salts to formazan derivatives by functional mitochondria [32,33]. Although redox sensitive viability assays have some limitations depending on the chemical nature of the compounds to be evaluated [45] they are widely used for measuring cell viability following nitrosative or oxidative stress [22,46,47]. The suitability of this method was furthermore documented by a previous study analyzing the effects of the spontaneous NO-donor DETA/NO on the viability of PCa-cells. Comparison of MTT with three commonly used methods to measure cell proliferation, i.e quantification of intracellular ATP, trypan blue dye exclusion and neutral red assay were found to generate similar results [22].

**Figure 7:**
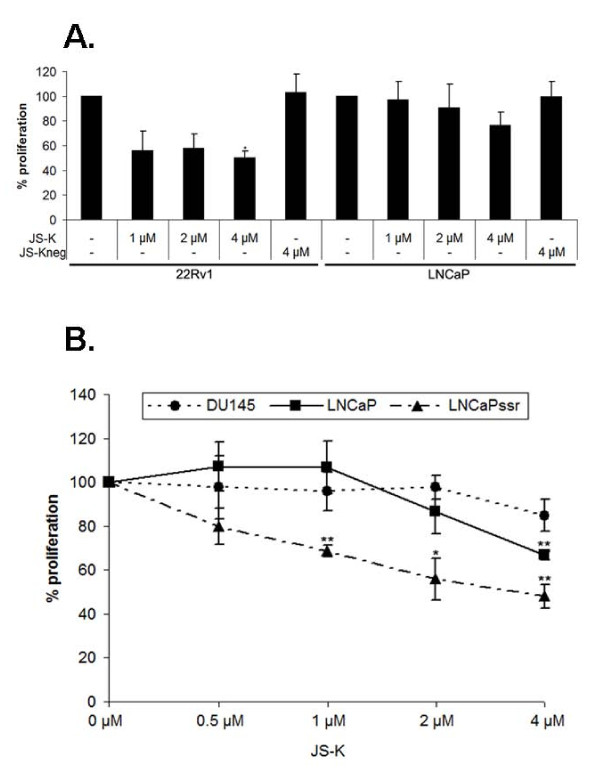
**Effects of JS-K on the proliferation of prostate cancer cells**. (A) 22Rv1 cells are more susceptible to the growth inhibitory effects of JS-K than the androgen sensitive LNCaP cells Cells were cultured in the absence/presence of increasing concentrations of JS-K (1,2,4 μM) or JS-K_neg _(4 μM). Cell viability was determined after 96 hours by a colorimetric MTT assay. Results are mean values of four independent experiments performed in quadruplicates. Data are presented as % of untreated controls ± SD, *p < 0.05. (B) Effects of JS-K on the AR negative DU-145 and the AR-positive LNCaP (hormone sensitive) and LNCaP-SSR (castration resistant). Cells were cultured in the absence/presence of increasing concentrations of JS-K (1, 2, 4 μM) or JS-K_neg _(4 μM). Cell viability was determined after 96 hours by a colorimetric MTT assay. Results are mean values of four independent experiments performed in quadruplicates.

A first series of experiments indicated that castration resistant 22Rv1 cells were more susceptible to the growth inhibitory effects of JS-K than the androgen sensitive LNCaP cells (Figure 7A). Maximal growth inhibitory effects were achieved at a concentration of 4 μM JS-K for 22Rv1 (growth inhibition 50 ± 6%, p < 0.05) and 4 μM JS-K for LNCaP (growth inhibition 23 ± 9%).

Given the highly divergent nature of the AR-positive cell lines 22Rv1 and LNCaP we repeated the proliferation experiments using the androgen sensitive LNCaP and LNCaP-SSR, an AR-positive castration resistant LNCaP-subline. The AR-negative DU-145 served as control (AR off-target effects). Interestingly, the castration resistant LNCaP-SSR cells were more susceptible to the growth inhibitory effects (growth inhibition at 1 μM JS-K 31 ± 2%, p < 0.01) than the parental LNCaP cells (growth inhibition at 4 μM JS-K 33 ± 1%, p < 0.01) (Figure 7B). In contrast to the AR-positive LNCaP and LNCaP-SSR the AR-negative DU-145 were less susceptible to the growth inhibitory effects of JS-K (growth inhibition at 4 μM JS-K 17 ± 9%).

## Discussion

Current treatment for advanced PCa is mainly based on androgen ablation therapies like chemical or surgical castration or application of anti-androgens. Although hormonal therapy is initially very effective, the benefit of androgen withdrawal is only transitory. While still expressing high levels of functional AR, some PCa cells acquire the ability to grow and survive under castrate levels of circulating androgens. The mechanisms involved in an altered AR-signalling of CRPC cells include an over-expression/amplification of the AR (hypersensitive pathway), point mutations broadening ligand-specificity of the receptor (promiscuous pathway) as well as activation of the AR by peptide growth factors or cytokines (outlaw pathway) [17]. A recently identified mechanism enabling CRPC cells to bypass the requirements for physiological levels of androgens is the expression of C-terminal truncated, constitutively active AR splicing variants termed AR-V. Due to the loss of the C-terminus, AR-Vs are lacking essential parts of the ligand binding domain [21]. In the absence of androgens, AR-Vs were shown to induce AR-signalling either as AR-V/AR-V-homodimers or AR-V/AR-heterodimers [18,48]. Whereas AR-V/AR-V homodimers are unaffected by hormone ablation in general, some AR-V/AR-heterodimers were insensitive to the commonly used anti-androgens [48]. Given the fact that both, AR wild type as well as AR-V, play a crucial role in a high percentage of CRPC there is an urgent need for an AR-blockade that does not involve the LBD.

Nitric oxide, a free radical gas, has been repeatedly shown to impair the function of nuclear receptors via targeting their cys_4_-type zinc fingers, located in the DBD of these receptors [49]. This is well exemplified by studies showing a NO-induced nitrosation of the cystein residues within the zinc finger structures of steroid receptors like the estrogen receptor (ER) [50]. As the DBD is a crucial part of functional full length AR as well as constitutively active AR-Vs, it is tempting to speculate that NO-donors able to release NO in a spatial and timely controllable manner could be of special interest for the treatment of deregulated AR-signalling in CRPC cells.

Inhibitory effects of NO on AR-function in PCa cells was first described *in vitro *using the spontaneous NO-donor Deta/NO [22]. In a recent clinical study Siemens et al. were able to show an increase of the PSA doubling time in patients treated with the NO-donor glyceryl trinitrate (GTN) [51]. No cardiovascular toxicities or serious side effects were encountered during treatment with GTN [51]. Although the number of patients enrolled in the study was relatively low (29 patients), our data and the clinical data with GTN [22,51] support the assumption that NO might be suitable for the treatment of advanced PCa.

Commonly used NO-Donors like Deta/NO, spontaneously dissociate in a pH dependent process thereby releasing the free radical NO. Unfortunately NO exhibits only a relatively short half-life in the tissue culture medium or body fluids. Therefore, we tested a potent NO-prodrug, the non-ionic nitroaromatic diazeniumdiolate JS-K. The compound was shown to be activated by glutathione S-transferase (GST), a key phase II detoxification enzyme that is frequently over-expressed in cancer tissue. Among the various isoforms of GSTs which are located in the cytoplasm and the mitochondria, mainly the π isoform is able to form the so called < Meisenheimer complex >[52] which finally delivers NO directly within the cell. First experimental *in vivo *studies using tumor xenografts in a mouse model, showed that JS-K exhibits a strong anti-tumor activity and is generally well tolerated by the host organism [5,23,24].

In order to test the effects of JS-K on AR signalling, we treated the AR-positive CRPC cell line 22Rv1 grown in presence of DHT with increasing concentrations of JS-K. Generation of intracellular NO was monitored indirectly by detection of nitrotyrosine using fluorescence microscopy. JS-K was able to inhibit the genomic functions of the AR in 22Rv1 cells. The inhibition of AR-signalling was neither due to an inhibition of AR-dimerization nor to an inhibition of nuclear translocation. Interestingly, JS-K was able to diminish intracellular levels of all AR-isoforms in PCa cells. However, in contrast to the dramatic downregulation of AR-V and AR in 22Rv1 cells the intracellular AR levels of LNCaP were only slightly affected.

So far, studies on intracellular steroid receptor levels following NO-treatment are sparse. In a previous study using Deta/NO as an NO-donor the concentration of the endogenous AR in LNCaP remained unaffected [22]. In contrast, DETA/NO was able to downregulate the intracellular levels of the Drosophila *melanogaster *ecdysteroid receptor (EcR) when overexpressed in chinese hamster ovary cells, CHO-K1 [53]. Although the heterologeous expression of an insect steroid receptor in CHO-K1 cells differs largely from the situation found in LNCaP cells that express high levels of intracellular AR under physiological conditions the present data suggest that there are cell specific differences in the sensitivity of cells towards NO-treatment. Moreover, the generation of NO by JS-K largely depends on the intracellular GST-activity that differs between different cell lines or cell types.

The fact that NO is able to inhibit the canonical WNT-pathway in different tumor cells [42-44] including prostate cancer cells (as seen in this study) offers a more intriguing explanation for the massive down regulation of AR and AR-V in 22Rv1 cells. There is experimental evidence that the WNT/β-catenin-pathway is able to up-regulate AR-mRNA expression in prostate cancer cell lines through interaction with TCF/LEF binding sites situated in the promoter region of the AR [41]. In contrast to the highly aggressive castration resistant 22Rv1 cells which are able to drive a WNT-typical T-cell factor (TCF)-dependent reporter gene activity, the androgen sensitive LNCaP are unable to do so [27]. In consequence, the inhibition of the canonical WNT-pathway would lead to a decrease of AR and AR-V mRNA in 22Rv1 cells but not or to a lesser extent in LNCaP.

In proliferation assays castration resistant 22Rv1 and LNCaP-SSR cells were more susceptible to the growth inhibitory effects of JS-K than LNCaP. Whereas LNCaP cells largely depend on androgenic stimuli, the 22Rv1 and LNCaP-SSR cells are able to grow under androgen deprived conditions. LNCaP cells express a full length AR with a point mutation at position 877 (T877A), enabling the AR to be stimulated by different steroids (promiscuous AR) [39]. In contrast to the hormone dependent LNCaP, the castration resistant LNCaP-SSR cells were shown to exhibit high levels of nuclear AR in the absence of hormonal stimuli [26]. Increased nuclear AR-levels were paralleled by elevated PSA-levels suggesting that the AR is active in these cells [26]. In 22Rv1 cells two AR-forms can be found: A larger AR-form expressing 3 zinc finger motifs due to the duplication of exon 3 (AR^Ex3dup^) and a C-terminally truncated, constitutively active AR-V [20]. In the absence of androgenic stimuli, some AR-Vs have been shown to form constitutively active AR-V-homodimers or AR/AR-V-heterodimers, thereby uncoupling the need of CRPC cells for physiological levels of androgens [18,48]. Although it is unknown whether AR^Ex3dup ^is able to form androgen independent heterodimers with AR-V in 22Rv1 cells, its expression of 3 zinc finger structures makes it probably also more susceptible to the effects of NO. The fact that NO targets the zinc finger structures of wild type AR, mutated AR-forms as well as AR-Vs suggests that NO-donors are promising compounds for the treatment of deregulated AR-activity. Moreover, the observation that CRPC cells like 22Rv1 or LNCaP-SSR, expressing either constitutively active AR-Vs or functionally deregulated full length AR, are more susceptible to the effects of JS-K than LNCaP cells further supports this assumption.

## Conclusions

From our *in vitro *studies we conclude that GST-activated NO-prodrugs like JS-K show features which might be suited for the treatment of advanced PCa. Therapies targeting the AR and the canonical WNT pathway may lead to a more efficient treatment of CRPC. Therefore, compounds like JS-K may serve as leader compounds for the development of NO-based therapeuticals, targeting key structures in castration resistant prostate cancer cells.

## Competing interests

The authors declare that they have no competing interests.

## Authors' contributions

ML, K-D S and MVC conceived the study; ML, WS, AH performed the experiments, MS AJS contributed to data analysis; MVC, K-D S, AJS, MS wrote the paper. All authors read and approved the final manuscript.

## Pre-publication history

The pre-publication history for this paper can be accessed here:

http://www.biomedcentral.com/1471-2407/12/130/prepub

## Supplementary Material

Additional file 1 **Figure 1S: JS-K does not inhibit AR-dimerization**. Complementary data to experiment presented in Figure 3: AR-dimerization was determined in DU-145 cells using the CheckMate Mammalian Two-Hybrid System described in Material and Methods. Results are mean values of three independent experiments performed in quadruplicates. (DOC 20 kb). Click here for file

Additional file 2 **Figure 2S: NO does not influence hormone-induced nuclear translocation of AR-EosFP in DU-145**. Complementary data to experiment presented in Figure 4. (TIFF 1541 kb). Click here for file
