# Supplementary figures and images for: JS-K, a glutathione/glutathione S-transferase-activated nitric oxide releasing prodrug inhibits androgen receptor and WNT-signaling in prostate cancer cells
Source: BMC Cancer. 2012 Mar 30;12:130. doi: 10.1186/1471-2407-12-130 (PMC3376035; doi:10.1186/1471-2407-12-130)

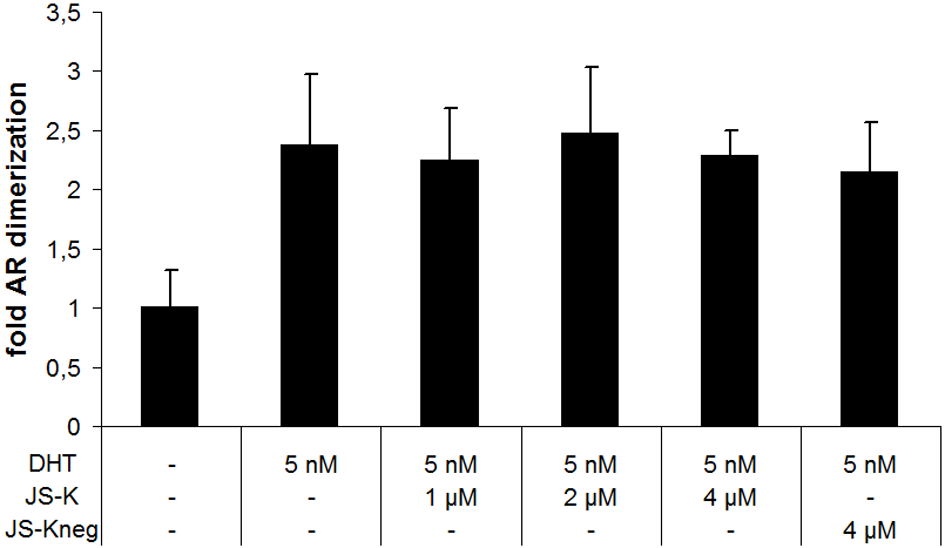

Supplement: Additional file 1 — Figure 1S: JS-K does not inhibit AR-dimerization. Complementary data to experiment presented in Figure 3: AR-dimerization was determined in DU-145 cells using the CheckMate Mammalian Two-Hybrid System described in Material and Methods. Results are mean values of three independent experiments performed in quadruplicates. (DOC 20 kb). [file 1471-2407-12-130-S1.TIFF]

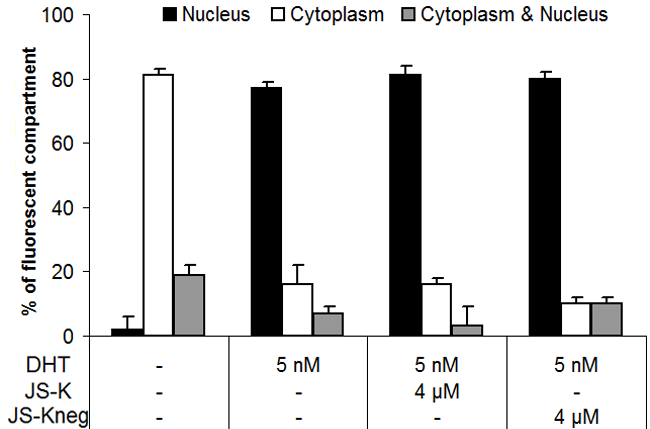

Supplement: Additional file 2 — Figure 2S: NO does not influence hormone-induced nuclear translocation of AR-EosFP in DU-145. Complementary data to experiment presented in Figure 4. (TIFF 1541 kb). [file 1471-2407-12-130-S2.TIFF]
